# Risk factors of primary angle-closure glaucoma: An exposome-wide association study in the China Kadoorie Biobank

**DOI:** 10.1016/j.apjo.2026.100378

**Published:** 2026

**Authors:** Zilun Shao, Shuo Gong, Charles Li, Canqing Yu, Pei Pei, Ling Yang, Yiping Chen, Enzhong Jin, Ting Shen, Jie Yu, Bin Wang, Zhi Chen, Ruoyan Wei, Jian Wang, Xiaoming Yang, Junshi Chen, Zhengming Chen, Xingtao Zhou, Yongyue Wei, Jun Lv, Liming Li, Huaidong Du, Dianjianyi Sun, on behalf of the China Kadoorie Biobank Collaborative Group

**Affiliations:** a Department of Epidemiology & Biostatistics, School of Public Health, Peking University, Beijing, China; b Clinical Trial Service Unit & Epidemiological Studies Unit (CTSU), Nuffield Department of Population Health, University of Oxford, United Kingdom; c Peking University Center for Public Health and Epidemic Preparedness & Response, Beijing, China; d Key Laboratory of Epidemiology of Major Diseases (Peking University), Ministry of Education, Beijing, China; e Department of Ophthalmology, Peking University People's Hospital, Beijing, China; f Beijing Key Laboratory of Ocular Disease and Optometry Science, Peking University People’s Hospital, Beijing, China; g Eye Center, The Second Affiliated Hospital, School of Medicine, Zhejiang University, Hangzhou, Zhejiang, China; h Department of Ophthalmology, Zhejiang Provincial People’s Hospital, People’s Hospital of Hangzhou Medical College, Hangzhou, Zhejiang, China; i Department of Ophthalmology, Tong De Hospital of Zhejiang Province, Hangzhou, Zhejiang, China; j Department of Ophthalmology and Vision Science, Eye & ENT Hospital of Fudan University, Shanghai, China; k Shanghai Medical College and Zhongshan Hospital Immunotherapy Translational Research Center, Shanghai, China; l NCDs Prevention and Control Department, Pengzhou CDC, Sichuan, China; m China National Center for Food Safety Risk Assessment, Beijing, China; n State Key Laboratory of Vascular Homeostasis and Remodeling, Peking University, Beijing, China

**Keywords:** Primary angle-closure glaucoma, Risk factors, Prospective cohort study

## Abstract

**Purpose:**

Primary angle-closure glaucoma (PACG) is a leading cause of irreversible blindness in Asia. Although traditionally considered an anatomical ocular disorder, the contribution of systemic metabolic, vascular, and lifestyle factors to PACG development remains unclear.

**Methods:**

Using data from the China Kadoorie Biobank (CKB), involving 499,102 participants aged 30–79 years at baseline, we explored associations of 117 exposures, including socio-demographics, lifestyle patterns, medical history, and physical measurements, with probable PACG (prob PACG) incidence. Elastic-Net, XGboost, and Cox regression methods were employed.

**Results:**

Over a median follow-up of 15.7 years, 3266 incident prob PACG cases were ascertained. After correcting for multiple testing, 26 factors were independently associated with prob PACG. Age, female sex, and postmenopausal status showed the strongest positive associations with pro PACG. Notably, several markers of metabolic and vascular ageing—including diabetes, higher random plasma glucose, larger waist circumference, and elevated pulse pressure—were independently associated with higher risk, whereas BMI was inversely associated (all *P < 0.01*). In addition, daily intake of dairy products and daily preserved vegetables, and peptic ulcer were associated with a higher risk, while higher physical activity and weekly tea consumption were associated with lower risk. No significant associations were found for hypertension, SBP, or DBP with prob PACG.

**Conclusions:**

Our findings suggest that PACG may be influenced not only by anatomical susceptibility but also by broader metabolic, vascular, and lifestyle factors. The clustering of several strong associations around markers of metabolic and vascular ageing provides new insights into PACG aetiology and may help identify priorities for future studies of risk stratification and prevention.

## 1 Introduction

Glaucoma is the leading cause of irreversible blindness globally, posing a formidable challenge to maintaining functional independence and quality of life in ageing populations.^1^ This chronic, progressive optic neuropathy is characterized by retinal ganglion cell degeneration and optic nerve damage, typically manifesting as peripheral vision deficits, which may subsequently progress to central vision impairment.^2^ Beyond mere visual impairment, the erosion of "visual healthspan" in older adults is intrinsically linked to heightened risks of falls, cognitive decline, and social isolation, thereby compromising the trajectory of healthy longevity. Epidemiological surveys from high-income countries indicate that fewer than 50% of individuals diagnosed with glaucoma through screening are aware of their condition. In low- and middle-income countries such as China, over 90% of glaucoma patients remain untreated, and up to 35% are already blind at the time of diagnosis.^3^ In 2010, approximately 8.4 million individuals were blinded by glaucoma, with over a quarter of these cases occurring in China.^4^ With global population ageing, the number of people affected by glaucoma is projected to rise to 112 million by 2040, with the greatest burden expected in African and Asian populations.^5^

A defining hallmark of glaucoma is its striking age-dependent manifestation, typically surfacing after the fifth decade of life. The exact causes and underlying mechanisms of glaucoma remain unclear. Well-established risk factors include intraocular pressure (IOP), age, sex, ethnicity, and family history.^2^ However, previous studies have not yet confirmed the impact of major modifiable exposures, including blood pressure,^6^, ^7^, ^8^ blood glucose,^9^ smoking,^10^ dietary patterns,^11^ and alcohol consumption,^12^ on glaucoma incidence. This highlights the need for a comprehensive exploration of a wide spectrum of potential risk factors for glaucoma through exposome-wide association studies. Additionally, most of the current evidence comes from Western populations, where primary open-angle glaucoma (POAG) is the predominant subtype.^13^ In contrast, primary angle-closure glaucoma (PACG), which is a distinct phenotype from POAG,^14^ is far more prevalent in China and other Asian countries, with incidence rates more than three times higher than those observed in Western populations.^15^ Despite the higher burden of PACG in Asian populations, research on this glaucoma subtype remains even more limited. Previous evidence has only focused on non-modifiable risk factors, including age, sex, ethnicity, ocular biometry, and genetics.^16^^,^^17^ However, these factors fail to explain the population differences in PACG prevalence and incidence in Chinese and other Asian populations.

While PACG has traditionally been regarded as a disease primarily driven by ocular anatomical characteristics, several studies have suggested that systemic metabolic and vascular factors may also influence glaucoma susceptibility.^15^ However, evidence remains fragmented and has largely focused on primary open-angle glaucoma, leaving the contribution of these factors to PACG poorly understood, particularly in Asian populations where disease burden is highest. To fulfil these knowledge gaps, we performed the current study to prospectively investigate the associations between a broad range of exposures and incident PACG, using data from the China Kadoorie Biobank (CKB)—a large nationwide prospective cohort study spanning ten geographically diverse regions in China.

## 2 Methods

### 2.1 Study design and participants

Details of the CKB study design and survey methods have been previously described.^18^ In brief, the CKB is a nationwide prospective cohort study that enrolled 512,724 participants, aged 30–79 years, from 5 urban and 5 rural areas across China between 2004 and 2008. At baseline, trained staff conducted face-to-face interviews using an electronic questionnaire to collect detailed information on sociodemographic factors, lifestyle characteristics (e.g., smoking, diet, and physical activity), and medical history. Physical measurements (e.g., blood pressure and anthropometry) were obtained using standardized protocols.^18^ Subsequent resurveys were conducted every 5–6 years after the baseline survey (i.e., in 2008, 2013–14, and 2021–22 respectively for the first three resurveys conducted thus far), in randomly selected subsets comprising 5–6% of surviving participants. These resurveys followed similar procedures to those used at the baseline survey, plus some new enhancement data collection. During the third resurvey conducted in 2021–22 (n = 25,087), ophthalmic data were collected for the first time in the CKB, including self-reported histories of eye diseases (glaucoma, cataract, and age-related macular degeneration), as well as ophthalmic measurements (visual acuity, IOP, and fundus photography).^19^^,^^20^ The study protocol for baseline survey, resurveys, and long-term follow-up was approved by the Ethics Review Committee of the Chinese Centre for Disease Control and Prevention (Beijing, China; 005/2004), Ethics Committee of Fu Wai Hospital (NO. 2018–1127), Peking University Institutional Review Board (IRB00001052–18097) and the Oxford Tropical Research Ethics Committee, University of Oxford (Oxford, UK; 025–04). All participants provided written informed consent prior to participation.

### 2.2 Intraocular pressure measurement and fundus photography in the CKB 3rd resurvey

IOP was measured at least six times for each eye (without glasses or contact lenses), using a handheld iCare IC100 tonometer. This tonometer measures the rebound motion caused by the impact of a probe on the cornea at a fixed distance of 4–8 mm (iCare Finland Oy). The device reports the mean value of four randomly selected satisfactory measurements as the final IOP value for each eye. In the current analysis, the highest IOP value across both eyes was taken as the participant's final IOP.

Fundus images were captured in a dark room using a handheld Optomed Aurora fundus camera. At least one fundus image per eye was captured for each participant, resulting in a total of 57,356 images. These images were evaluated and interpreted by three ophthalmologists (practicing at top-tier hospitals in China) in conjunction with artificial intelligence (AI) analysis.^21^ Diagnoses of glaucoma suspect and increased cup-to-disc ratio were made based on consensus between ophthalmologists and the AI system. Additionally, AI-assisted readings provided quantitative measurements, including cup diameter (horizontal and vertical), disc diameter (horizontal and vertical), cup-to-disc ratio (horizontal and vertical), optic disc rim width (superior, inferior, nasal, and temporal), as well as optic disc area, cup area, rim area, and their respective area ratios. These ophthalmic measurements were solely used to select participants with no identified evidence of glaucoma for the control group in the nested case-control component. We did not use these measurements to ascertain prob PACG or to add cases to the outcome definition in the full CKB cohort.

### 2.3 Exposures

This study examined 12 distinct categories of exposures that may be potential risk or protective factors, encompassing 117 variables, as follows: (1) sociodemographic factors (n = 12 variables), (2) tea consumption (n = 5), (3) alcohol consumption (n = 5), (4) tobacco use history (n = 3), (5) dietary factors (n = 20), (6) passive smoking and indoor air pollution (n = 4), (7) personal and family medical history (n = 37), (8) physical activities (n = 2), (9) female reproductive history (n = 5), (10) sleep patterns, psychological status, and mental health (n = 8), (11) physical examination (n = 14), and (12) blood tests (n = 2). A full classification scheme is provided in the appendix (Table S1), while detailed information about data collected in the CKB study could be found at https://www.ckbiobank.org/.

### 2.4 Follow-up and ascertainment of incident outcomes

After completing the baseline survey, participants were continuously monitored for mortality and hospitalization through electronic linkages to national death and major chronic disease registries, as well as health insurance databases and the initial pages of hospitalization records, which cover > 97% of all hospitalization episodes.^22^ All recorded events were encoded using International Classification of Diseases, Tenth Revision (ICD-10) diagnostic codes by trained staff who were blinded to the participants' baseline characteristics. Incident PACG outcome was defined as the first recorded PACG hospitalization across all available data sources. The distribution of PACG (plus other glaucoma subtypes) and data sources across the entire CKB baseline population (i.e., prior to the application of any inclusion or exclusion criteria) is presented in the appendix (Table S2). Given that PACG is the major subtype of glaucoma in China,^23^ particularly in hospitalization records,^15^ we defined PACG in this study as “probable PACG (prob PACG)” using ICD-10 codes H40.0 (glaucoma suspect), H40.2 (PACG), or H40.9 (unspecified glaucoma) to maximize the number of events and therefore statistical power. The validity of this proxy phenotype mapping in the CKB cohort has been explicitly evaluated and confirmed by our concurrent genomic validation study.^24^ Specifically, polygenic risk score (PRS) analyses demonstrated that these unspecified glaucoma cases in the CKB shared a homogeneous genetic architecture exclusively with PACG (OR per SD increase: 1.32, 95% CI: 1.08–1.61) rather than POAG, justifying the consolidation of these codes into a single 'prob PACG' outcome. Sensitivity analyses were conducted with a more specific outcome definition of "definite PACG" (ICD code: H40.2). Participants who did not develop the outcome of interest were censored at the time of death, loss to follow-up, or on December 31, 2022, whichever came first.

### 2.5 Statistical analysis

Baseline characteristics were summarized as standardized means or proportions, adjusted for age, sex, and region using multiple linear regression or logistic regression, as appropriate. For continuous variables, standard deviations were also reported.

To overcome the potential under-ascertainment of glaucoma in CKB, which collected only hospitalized events during follow-up, a multi-phase statistical approach was used to screen for potential factors associated with prob PACG incidence (Fig. 1). Firstly, a nested case-control study was established (*Step 1*), with 3266 ICD-10-coded cases, identified from the entire CKB cohort (n = 512,724), included in the case group. The control group (i.e., non-glaucoma, n = 13,339) was selected from the participants attending the 3rd resurvey of CKB, using the following criteria: (1) no reported eye diseases during the entire follow-up period (i.e., ICD-10: H00-H59); (2) no self-reported history of eye diseases during the 3rd resurvey; (3) measured IOP < 21 mmHg for both eyes; (4) vertical cup-to-disc ratio (VCDR) ≤ 0.5 for both eyes; and (5) features of fundus images do not suggest the presence of glaucoma (Figure S1). Secondly, feature selection (*Step 2*) using Elastic-Net and XGboost (Supplementary Methods 1–2) was conducted in a discovery stage to identify factors potentially associated with incident prob PACG, from a total of 117 candidate variables covering 12 above-described categories. Finally, in a subsequent validation stage (*Step 3: a full-cohort association analysis*), selected factors in the above Step 2 were assessed in the full CKB cohort, using Cox proportional hazards regression models with age as the time scale, to estimate their associations with incident prob PACG. Confounders were included in the models according to established epidemiological principles and prior evidence (e.g., age, sex and region) suggesting that they may influence the exposure, the outcome, or both.^2^ For continuous exposure variables, we used restricted cubic splines (RCS) with three knots in Cox models to flexibly model their associations with incident prob PACG. For each exposure, observations outside the 2.5th and 97.5th percentiles were excluded before fitting the spline models, with three knots placed at the 10th, 50th, and 90th percentiles of the exposure distribution in the remaining analytic sample. Non-linearity was assessed using likelihood ratio tests comparing models with and without cubic spline terms. Additionally, sensitivity analyses were conducted to assess the robustness of our findings: (1) using only definite PACG cases (ICD-10: H40.2) as the outcome; (2) accounting for death as a competing risk using the Fine–Gray subdistribution hazard model.^25^

**Fig. 1:**
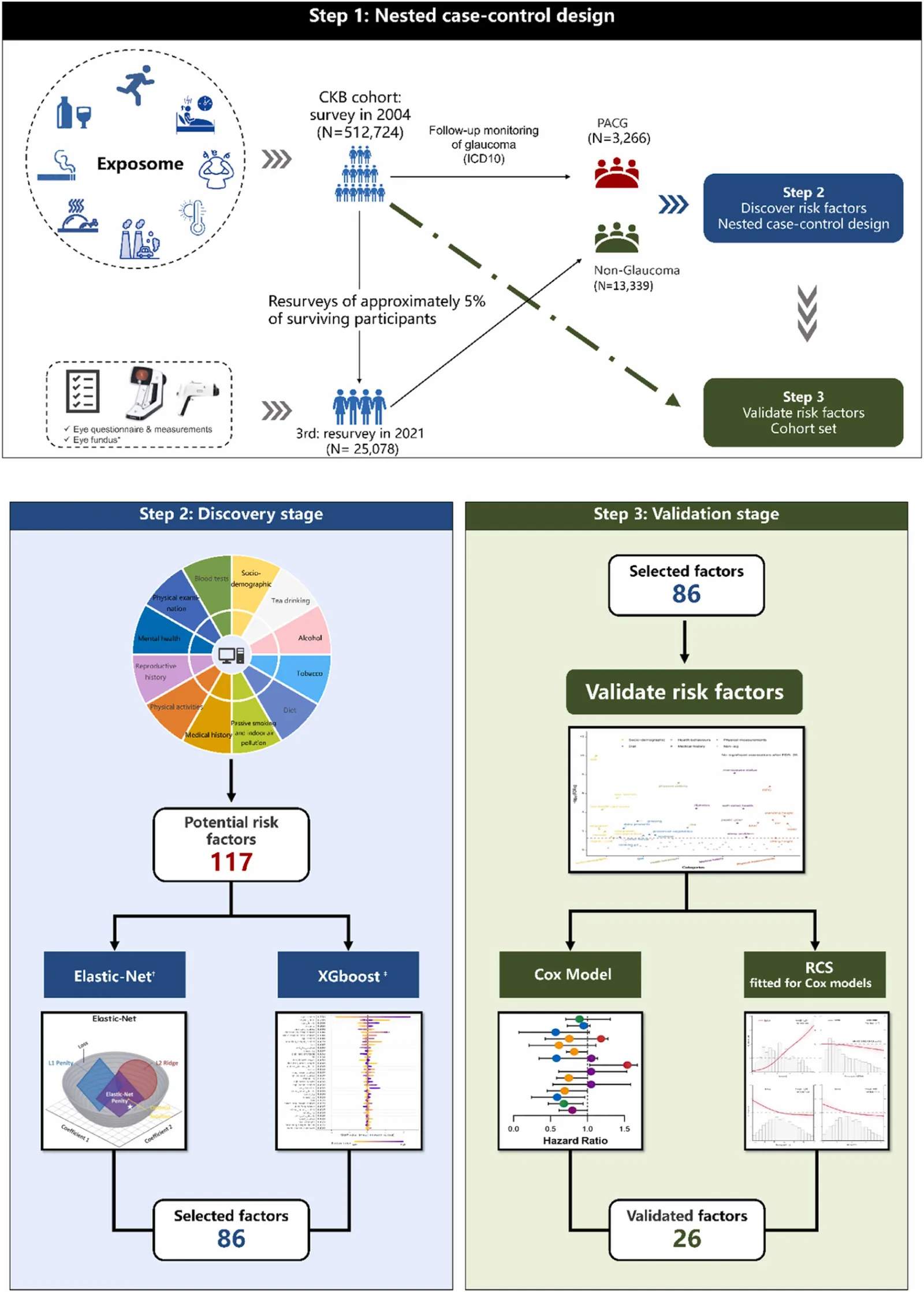
Summary of study design. *Eye fundus images were evaluated through dual-quality control and interpreted by three ophthalmologists in conjunction with AI-based analysis. ^†^Elastic-Net: (1) Applied L1 and L2 regularization simultaneously; (2) Performed a bootstrap procedure with 100 resampling iterations: for each iteration, the data were randomly split into training and validation sets. Penalized logistic regression was performed, and the coefficients of selected features were recorded; (3) Calculated the selection frequency of each feature across all iterations; features chosen in at least 80 of the 100 resampling iterations were retained. ^‡^XGboost: (1) Randomly split the data into training (80%) and test sets (20%); (2) Applied Bayesian optimization (100 iterations) to identify optimal hyperparameters for the XGboost model; (3) Evaluated model performance using the area under the ROC curve (AUC), with 10-fold cross-validation; (4) Selected the most important features based on Gain, retaining features that accounted for the top 85% of cumulative importance. Step 3 represents a full-cohort association analysis.

Statistical analyses were performed using either R version 4.3.1 (Elastic-Net, XGboost, Cox regression, and RCS), Python version 3.8.20 (Isolation Forest model for outlier detection), or SAS 9.4 (Fine–Gray subdistribution hazard model). Two-sided *P* values < 0.05 were considered statistically significant unless otherwise stated.

### 2.6 Role of the funding source

The funders had no role in study design, data collection, analysis, and interpretation, or writing of the report.

## 3 Results

### 3.1 Baseline characteristics and follow-up results

From the entire CKB cohort (n = 512,724), after excluding 13,622 participants with missing (n = 8580) or outlier (n = 5042) data, a total of 499,102 adults aged 30–79 years were included in the main analysis (*Step 3*) (Figure S2). During the median follow-up of 15.7 years (IQR 15.0–16.8), totaling 7,236,564 person-years, a total of 3266 prob PACG cases were recorded. Compared to individuals without prob PACG, those with prob PACG were more likely to be older, to be female, to reside in an urban area, to have lower educational attainment, to be retired, and to be non-farmers at baseline (Table 1).

**Table 1:** Baseline characteristics of study participants by prob PACG status.

	**Non- prob PACG** **(N = 495,836)**	**prob PACG** **(N = 3266)**	**All** **(N = 499,102)**
**Age, years (SD)**	51.5 (10.7)	57.7 (9.2)	51.5 (10.7)
**Age group, %**			
30–39	15.2	2.9	15.1
40–49	30.0	16.5	29.9
50–59	30.8	35.3	30.8
60–69	17.8	35.1	17.9
70–79	6.3	10.1	6.3
**Women, %**	59.1	69.4	59.1
**Urban, %**	44.7	59.4	44.8
**Highest education, %**			
No formal school	18.2	19.0	18.2
Primary school	32.2	33.3	32.2
Middle school	28.4	25.8	28.4
High school	15.3	14.9	15.3
College degree or above	5.9	7.0	5.9
**Occupation, %**			
Agriculture worker	41.2	27.3	41.1
Factory worker	14.4	7.9	14.3
Technical personnel	23.5	22.9	23.5
Retired	16.6	38.6	16.7
Unemployed	4.3	3.3	4.3
**Household income (yuan), %**			
< 5,000	9.6	7.4	9.6
5,000–10,000	18.4	14.8	18.4
10,000–20,000	29.1	32.8	29.1
20,000–35000	24.7	28.0	24.7
≥ 35000	18.2	17.1	18.2

### 3.2 Selection and validation of PACG risk factors

Using Elastic-Net analysis with bootstrapping, a total of 84 (out of 117) variables were identified as being associated with PACG. Separately, XGboost selection identified 31 associated variables; most of them were selected by the Elastic-Net analysis method as well. All variables identified by either of these two discovery approaches were included in the validation analysis (n = 86, Table S3). Following a stepwise Cox regression analysis, 26 variables—in five categories (sociodemographic factors, diet, health behaviours, medical history, and physical measurements)—remained statistically significant after False Discovery Rate (FDR) correction using the Benjamini-Hochberg method (Fig. 2). Among these 26 significant variables, 8 were associated with a reduced risk of incident PACG. The association of age (which was positively associated with PACG) had the smallest FDR-q value, followed by menopause status (positive), physical activity (inverse), random plasma glucose (positive), and female sex (positive), while the associations of occupation, region, cooking oil type used, having sleep problems, and sitting height with PACG were only borderline significant after correcting for multiple testing.

**Fig. 2:**
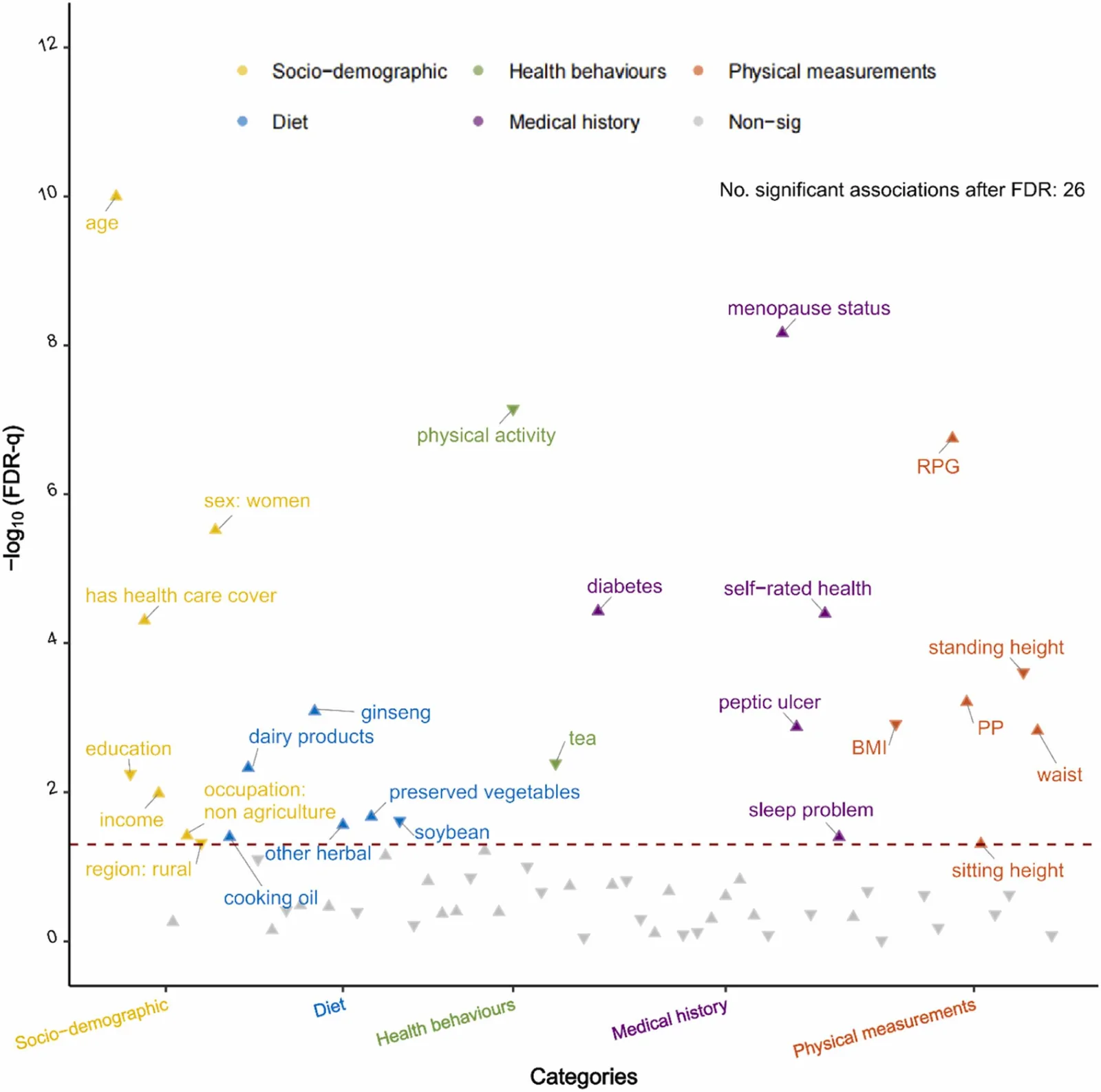
Exposome-wide analysis of factors potentially associated with prob PACG incidence. FDR, False Discovery Rate; FDR-q, FDR-adjusted p-value (Benjamini-Hochberg method); BMI, body mass index; RPG, random plasma glucose; PP, pulse pressure (PP, SBP -DBP); sleep problem, for a detailed definition, refer to Table S1. The y-axis represents the negative log (10) of the FDR-q (for categorical variables, the FDR-q refers to the significance for each category; for continuous variables, the FDR-q indicates the trend test). The horizontal red dashed line indicates the 0.05 cut-off. Upward triangles represent factors associated with an increased risk of prob PACG, while downward triangles represent factors associated with a reduced risk.

### 3.3 Associations of age, sex and region with PACG

The risk of PACG was higher at older age (HR= 1.07, 95% CI: 1.06–1.07 per 1 year older) and in women (1.35, 1.18–1.55) (Fig. 3**a**), but no clear difference was observed across 10 regions or between rural and urban areas (data not shown). Age showed a strong positive dose-response relationship with PACG risk, particularly after around 50 years of age (P for non-linearity <0.001) (Fig. 4). Compared to those aged less than 50 years, those aged 50–60 years had an approximately 2.7-fold higher hazard of PACG (2.68, 2.41–2.97), and those aged over 70 years had a risk more than four times higher (4.50, 3.83–5.28).

**Fig. 3:**
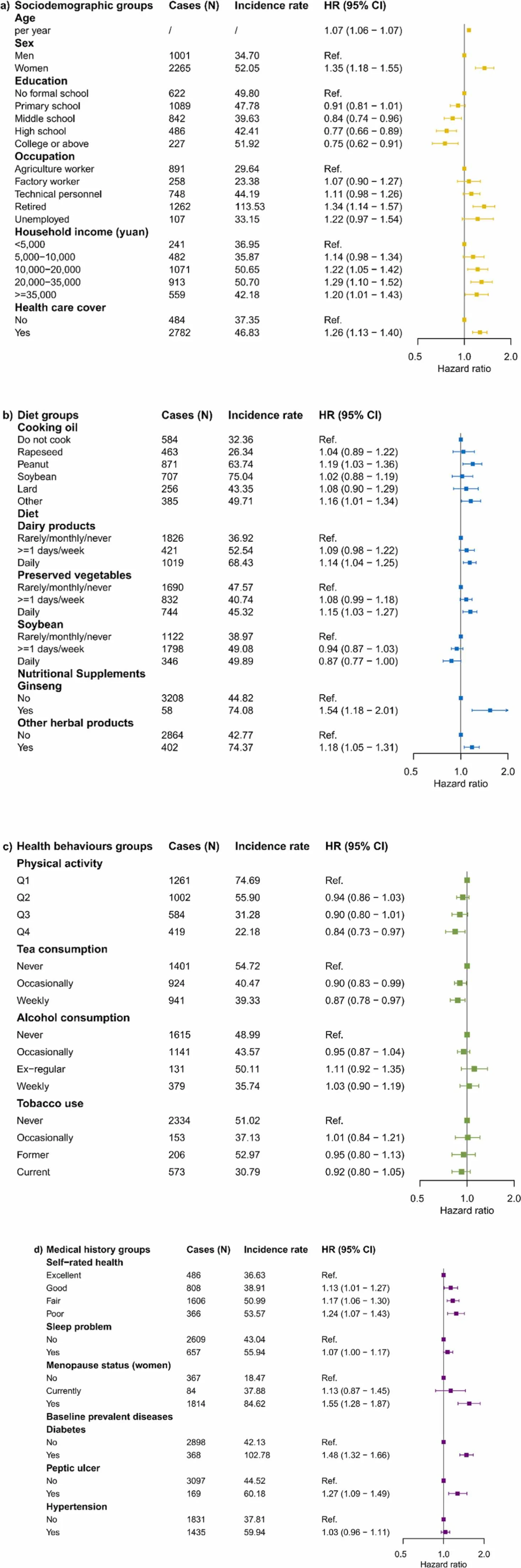
Associations between selected exposures and prob PACG incidence. HR= Hazard ratio; Ref= Reference; Incidence rate: 100,000 person-years; PA= Physical activity: The quartiles of physical activity were determined separately for men and women. Model adjustments were performed using a stepwise regression strategy. For sociodemographic factors, Model 1 was adjusted for age, sex, region, education, occupation, marital status, household income, and healthcare coverage. Model 2 additionally adjusted for dietary factors and retained significant variables as the final Model 2. Model 3 additionally adjusted for health behavior factors and retained significant variables as the final model 3. Finally, Model 4 additionally adjusted for medical history factors and retained significant variables as the final model [Model 1 =age+ sex+ region+ education+ occupation+ marital status+ household income+ healthcare coverage; Model 2 = Model1 + dairy+ preserved vegetables+ soybean+ ginseng+ herbs+ cooking oil; Model 3 = Model2 + pa+ tea+ smoking+ drinking; Model4 = Model3 + sleep problem+ self-rated health+ menopause status (only in women)+ diabetes+ peptic ulcer+ hypertension].

**Fig. 4:**
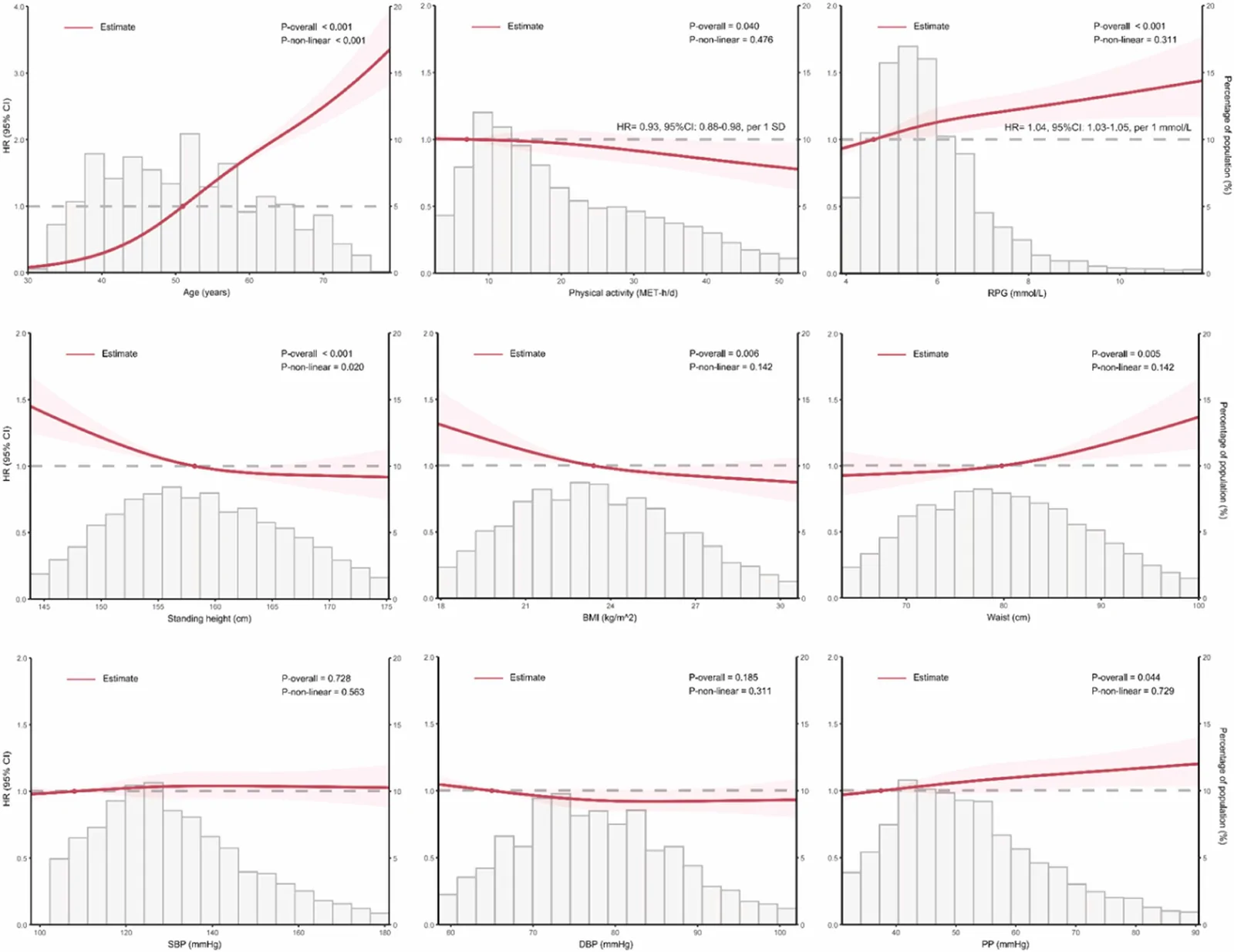
Associations between continuous exposures and prob PACG incidence. HR = hazard ratio; CI = confidence interval; RPG= random plasma glucose; MET-h/d = Metabolic equivalents of task per hour per day; SBP = systolic blood pressure; DBP = diastolic blood pressure; PP = pulse pressure (PP= SBP -DBP); BMI = body mass index. All analyses used restricted cubic splines and adjusted for age (except for the model, which examined age as the main exposure), sex, region, education, occupation, marital status, household income, healthcare coverage, tea consumption, dairy products, preserved vegetables consumption, soybean intake, tobacco, and alcohol consumption. For blood pressure analyses, additional adjustment was made using the residuals of SBP/DBP after regressing DBP on SBP (and vice versa); for adiposity measures, BMI and waist circumference were mutually adjusted. For each continuous exposure, restricted cubic spline models used three knots located at the 10th, 50th, and 90th percentiles of the exposure distribution after exclusion of values outside the 2.5th and 97.5th percentiles. Hazard ratios were calculated relative to the 10th percentile (PA, RPG, SBP, DBP and PP) or the 50th percentile (age, standing height, BMI, and waist) of the corresponding exposure.

### 3.4 Associations of categorical exposures with PACG incidence

After adjusting for age, sex, study region, education, occupation, marital status, household income, and healthcare coverage status where appropriate, having healthcare coverage was associated with 26% (1.26, 1.13–1.40) higher risk of PACG (Fig. 3**a**). A higher level of education was associated with a lower risk of PACG, with individuals in the highest education category (i.e., college education or higher) having an HR of 0.75 (0.62–0.91) compared to those with no formal education. Compared with agricultural workers, retirees (1.34, 1.14–1.57) had a higher risk of prob PACG.

Among the 20 dietary factors, six were significantly associated with PACG incidence (Fig. 3**b**). Compared with individuals who did not cook, those who used peanut oil had a significantly higher risk of PACG (1.19, 1.03–1.36), while no significant association was found for groups using rapeseed, soybean, lard, or other cooking oils. Higher consumption of dairy products was associated with a higher risk of PACG (*P*_*trend*_ < 0.001), with daily intake being associated with a 14% higher risk (1.14, 1.04–1.25). Similarly, those who consumed preserved vegetables daily had a 15% higher risk of PACG (1.15, 1.03–1.27). In contrast, soybean consumption was inversely associated with PACG risk (*P*_*trend*_ = 0.02), although the HR for daily consumers, in comparison with non-consumers, was only borderline significant (0.87, 0.77–1.00). Among the five nutritional supplements investigated, only taking ginseng and other herbal products were significantly associated with a higher risk of PACG, with HRs being 1.54 (1.18–2.01) and 1.18 (1.05–1.31), respectively.

Among the additional lifestyle risk factors, including physical activity, tea consumption, alcohol consumption, and tobacco use, only physical activity and tea consumption were significantly associated with PACG (Fig. 3**c**). Being in the highest quartile of physical activity was associated with a 16% lower risk (0.84, 0.73–0.97) of PACG than those in the bottom quartile group. Individuals who drank tea either occasionally or regularly had a lower risk of PACG compared to those who never consumed tea, with HRs being 0.90 (0.83–0.99) and 0.87 (0.78–0.97), respectively. As for those health status indicators examined in our analysis, significant associations were observed for self-rated health, diabetes, peptic ulcer, and female menopause status. Compared to individuals who rated their health as excellent, those who rated it as good (1.13, 1.01–1.27), fair (1.17, 1.06–1.30), or poor (1.24, 1.07–1.43) had a significantly higher risk of PACG (Fig. 3**d**). Participants with diabetes had a substantially higher risk of PACG (1.48, 1.32–1.66). Similarly, individuals with a history of peptic ulcers had a higher risk of developing PACG (1.27, 1.09–1.49) as well. In the analysis restricted to women only, those postmenopausal women had a significantly higher risk of PACG (1.55, 1.28–1.87) compared to those who were premenopausal. Hypertension status was not significantly associated with PACG risk (1.03, 0.96–1.11).

### 3.5 Associations of continuous exposures with PACG incidence

As shown in Fig. 4, an inverse linear association was observed between levels of physical activity (MET-h/d) and PACG risk, with 1 SD higher levels of physical activity associated with a 0.93-fold lower PACG risk (95% CI: 0.88–0.98). A roughly linear positive association was observed between random plasma glucose (RPG) and PACG incidence, with each 1 mmol/L higher RPG associated with a 1.04-fold higher risk of PACG (95%CI: 1.03–1.05). Compared to those with RPG around 5.0 mmol/L, those in the highest RPG group (>11 mmol/L) had a 78% higher risk of PACG (95%CI: 1.50–2.11). We also found a strong L-shaped inverse relationship between standing height and PACG risk, i.e., a lower PACG risk in individuals with taller standing height, but this relationship became less clear at approximately 158 cm in the general population (165 cm in men and 155 cm in women). Similarly, we also found a negative trend in the association between BMI and PACG risk, with those having the highest level (≥28 kg/m ^2^) of BMI having 15% lower risk. However, waist circumference (WC) was in a significantly positive association with PACG, with those in the highest category of WC (>90 cm) having a 24% higher risk of PACG than those in the lowest category (≤70 cm). For blood pressure, although a positive trend was observed for SBP and a negative trend for DBP, neither reached statistical significance. On the other hand, pulse pressure (PP) showed a significant positive linear relationship with PACG risk, with each 10 mmHg higher PP being associated with a 4% higher risk.

In sensitivity analysis using definite PACG (ICD-10: H40.2, n = 1015) as the outcome, the results were essentially the same, despite some associations no longer being statistically significant due to reduced statistical power (Table S4**,** Figure S3). In addition, accounting for competing risk due to deaths did not essentially alter the main findings either (Table S5).

## 4 Discussion

In this population-based prospective cohort study, we systematically investigated a broad range of potential risk factors for incident PACG and identified 26 factors independently associated with PACG risk. Beyond confirming established risk factors such as older age (HR=1.07, 95% CI: 1.06–1.07 per year) and female sex (1.35, 1.18–1.55), a notable pattern emerged whereby many of the strongest associations clustered around markers of metabolic and vascular ageing, including diabetes, elevated plasma glucose, central adiposity, and increased pulse pressure. This pattern suggests that PACG may not be solely an anatomical disease of the anterior chamber angle, but may also reflect broader systemic ageing processes. While current understanding of PACG has largely focused on ocular biometric characteristics, genetics, and demographic factors, our findings support a more integrated model in which metabolic dysfunction, vascular ageing, and lifestyle behaviours jointly contribute to disease risk.

These findings raise the possibility that systemic metabolic and vascular processes may influence PACG susceptibility through mechanisms extending beyond anterior chamber anatomy alone. Metabolic dysregulation and vascular ageing may affect ocular perfusion, autonomic regulation, extracellular matrix remodelling, or lens and anterior segment characteristics, thereby modifying the likelihood of angle closure in anatomically susceptible eyes. This perspective extends current understanding of PACG, which has largely focused on ocular anatomy and genetic predisposition, and provides a useful framework for interpreting the associations observed in the present study.

Previous studies have mainly focused on total glaucoma or POAG, with very limited research specifically addressing the risk profiles of PACG. Therefore, we could not directly compare most of our findings with previous literature. However, the little existing evidence seems to suggest that age, sex, ethnicity, ocular anatomy parameters (i.e., intraocular pressure, anterior chamber depth, and axial length), body weight, cataract, hyperlipidemia, and genetic factors are the major determinants of PACG,^17^ consistent with our findings. For instance, age had the strongest association with PACG in our study, showing a sharp increase in PACG incidence after 50 years of age, which might be related to the age-related changes in lens thickness and anterior chamber depth contributing to angle narrowing.^15^ We also observed that women, particularly postmenopausal women, are at increased risk for PACG. This finding is consistent with previous evidence (mainly on POAG) suggesting a sex-specific anatomical predisposition and a potential hormonal protective effect, which may reduce retinal ganglion cell apoptosis and enhance biomechanical support of the optic nerve.^26^

We observed distinct associations between several different socio-economic factors and PACG risk, which have not been reported in previous studies, including the higher risk of PACG among retirees, those with higher household income, people with lower education level, and participants having healthcare coverage. Some of these associations (e.g., retirees having higher risk of PACG) might be related to residual confounding by age and existing health conditions such as poor vision, but the opposite direction of associations of income and education with PACG suggests that affordability to health care (i.e., income) and health consciousness (i.e., education) may be associated with PACG risk via different underlying mechanisms. For instance, better access to healthcare services could lead to earlier detection of PACG, leading to a positive association, while enhanced health literacy and consciousness could lead to health-promoting behaviors, thus reducing PACG risk.^27^

Several dietary factors were significantly associated with PACG risk in our study, suggesting different pathways/mechanisms might be involved in the aetiology of PACG, although further studies are needed to confirm/refute these speculations. For instance, intake of dairy products (a major source of dietary calcium) was positively associated with PACG risk in our study. Previous studies have found that high calcium intake was associated with increased glaucoma risk (highest quintile OR [95% CI]: 2.44 [1.25–4.76]), suggesting a potential role of calcium in glaucoma pathophysiology.^28^ Calcium is also known as an oxidant that plays a significant role in promoting oxidative stress and the degeneration of the trabecular meshwork and retinal ganglion cells.^29^ With regard to preserved vegetables, the positive association with PACG observed in our study might be attributed to the high sodium content in preserved vegetables, which has been associated with IOP and optic nerve cell apoptosis.^30^ This result is in line with recent findings from the UK Biobank suggesting that urinary sodium excretion, a biomarker of sodium intake, is a modifiable risk factor for glaucoma, particularly in genetically predisposed individuals.^31^ Finally, for the inverse association between soybean intake and PACG risk observed in our study, isoflavones in soya products may play a role because of their estrogenic and antioxidative properties.^32^

Our finding of an inverse association between physical activity and PACG risk aligns with previous studies showing that regular physical activity may reduce IOP and enhance ocular blood flow.^33^ Similarly, the inverse association of habitual tea consumption with PACG risk found in this study is consistent with those from other studies. For instance, a study using data from the U.S. National Health and Nutrition Examination Survey (NHANES) reported a 74% reduced risk of glaucoma in daily tea drinkers,^34^ much stronger than the association we observed in the current study (HR=0.87, 95%CI: 0.78–0.97). Polyphenolic compounds in tea are known for their antioxidative and neuroprotective effects, which may mitigate glaucomatous damage.^35^

Among the medical history factors investigated, diabetes showed a strong and consistent association with PACG risk. Additionally, RPG demonstrated a positive linear relationship with PACG risk, consistent with previous studies,^9^ which have implicated the role of blood glucose levels in glaucoma pathophysiology. A meta-analysis of 47 studies also found that individuals with diabetes had a 1.48-fold increased risk of glaucoma (95% CI, 1.29–1.71), and the risk increased by 5% for each year following diabetes diagnosis.^9^ In addition, our results also support that the prevalence of peptic ulcer is a risk factor for PACG. To the best of our knowledge no previous report of this novel association, but several studies in the literature have suggested that *Helicobacter pylori* infection may be associated with glaucoma.^36^ Growing interest in exploring the relationship between gut microbiota and glaucoma^37^ may generate novel knowledge about a link between digestive system diseases and glaucoma. Such development may help with identifying prevention and treatment targets for glaucoma and high-risk individuals to develop glaucoma.

Although not all, some previous studies reported an inverse association of height and BMI with glaucoma,^38^, ^39^, ^40^ similar to what we observed in the current study. For instance, the Beijing Eye study showed that taller body height was associated with a lower prevalence of glaucoma (β= −1.20, P = 0.03).^38^ In addition, a cross-sectional analysis of data from the All of Us study in the USA (n = 156,476 participants) reported that being overweight or obese, as defined using BMI, was associated with a 21% and 23% lower risk of glaucoma than those who were not overweight or obese.^39^ Similarly, a multicohort study (The PROGRESSA, CLSA, and UK Biobank) also found that a lower BMI was associated with a higher risk of glaucoma (OR= 0.94/SD; 95% CI: 0.91–0.98).^38^ Together, these findings may suggest that lighter and shorter individuals may have some developmental or anatomical characteristics—such as smaller ocular volume—that predispose individuals to narrow anterior chamber angles,^38^ thus being more vulnerable to develop PACG. On the other hand, the positive association between waist circumference and PACG supports a potential role of central obesity, which is more strongly related to insulin sensitivity than BMI, in PACG pathogenesis. However, two previous Mendelian randomization analyses reported a potential causal effect of central obesity on POAG development,^41^^,^^42^ but no such studies on PACG have been published.

The role of blood pressure on IOP regulation and glaucoma risk has been of interest for many years because systemic hypertension may contribute to increased IOP through either overproduction or impaired outflow of aqueous humor.^8^ However, the findings (mainly on POAG) have been controversial, with some studies reporting a positive association,^43^ while others do not.^44^^,^^45^ In our study, neither SBP nor DBP was significantly associated with PACG, but PP had a weak but significant positive association with PACG risk. Higher PP, as a marker of vascular ageing and arterial stiffness, may impair ocular blood flow and exacerbate optic nerve vulnerability, potentially contributing to glaucoma progression.

The current study is the first large-scale, prospective, population-based study on exposome-wide factors associated with PACG, but several limitations should be acknowledged. First, in our main analysis, we defined PACG cases as “prob PACG” (including glaucoma suspect and unspecified glaucoma) to maximize statistical power and offset the under-ascertainment of specific subtypes in early electronic health records. While this broad definition could theoretically introduce phenotypic misclassification (such as potential contamination by primary open-angle glaucoma [POAG]), multiple layers of clinical and genetic evidence rigorously support the robustness of our approach. Clinically, our sensitivity analyses restricted purely to definite PACG cases (ICD-10: H40.2) yielded essentially consistent risk profiles, despite attenuated statistical power. Genetically and etiologically, our phenotyping strategy is strongly cross-validated by an independent, large-scale multi-biobank study utilizing the CKB genomic dataset,^24^ in which unspecified glaucoma cases showed genetic and age-related patterns more consistent with PACG than with POAG. These findings provide additional support for the validity of the pro PACG phenotype used in the present analysis. Therefore, leveraging this consolidated phenotype for our exposome-wide screening is clinically and biologically justified, ensuring both high statistical efficiency and phenotype purity. Second, PACG ascertainment relied primarily on hospitalization records and may therefore have missed early or asymptomatic cases, preferentially capturing clinically diagnosed and more severe disease. Such under-ascertainment is likely to be largely non-differential with respect to the exposures examined and may have reduced statistical power and attenuated the observed associations. Third, although our analyses were adjusted for a wide range of potential confounders, residual confounding cannot be ruled out given the measurement errors in covariate variables and the existence of unknown confounders. Fourth, the use of the same population in both the detection and validation stages may lead to overfitting or inflate the associations between the identified factors and incident PACG. To minimize this potential bias, we carefully selected a control group that excluded individuals with a history of eye diseases or glaucoma during the follow-up period, and employed robust statistical methods. Nevertheless, future studies are needed to further confirm or refute our findings. Finally, during the baseline survey, we did not conduct ophthalmic measurements or collect information on prior eye diseases. This limitation restricted our ability to perform separate analyses comparing participants with and without certain eye conditions.

In summary, this large prospective study provides new evidence that PACG may be influenced not only by ocular anatomical susceptibility but also by broader metabolic and vascular ageing processes. The clustering of multiple factors around markers of metabolic dysfunction, vascular ageing, and healthier lifestyle behaviours supports a more integrated framework for understanding PACG development. These findings provide new insights into the aetiology of PACG and may help inform future strategies for risk stratification, prevention, and early intervention in populations at high risk of irreversible vision loss.

## Declaration of Competing Interest

No conflicting relationship exists for any author.

## Data Availability

The China Kadoorie Biobank (CKB) is a global resource for the investigation of lifestyle, environmental, blood biochemical and genetic factors as determinants of common diseases. The CKB study group is committed to making the cohort data available to the scientific community in China, the UK, and worldwide to advance knowledge about the causes, prevention, and treatment of disease. For detailed information on what data is currently available to open access users and how to apply for it, visit: https://www.ckbiobank.org/data-access. Researchers who are interested in obtaining the raw data from the China Kadoorie Biobank study that underpins this paper should contact pdc@kscdc.net. A research proposal will be requested to ensure that any analysis is performed by bona fide researchers and - where data is not currently available to open-access researchers - is restricted to the topic covered in this paper.
