# Supplementary material for: Risk factors of primary angle-closure glaucoma: An exposome-wide association study in the China Kadoorie Biobank
Source: Asia Pac J Ophthalmol (Phila). 2026 Sep-Oct;15(5):None. doi: 10.1016/j.apjo.2026.100378 (PMC13722677; doi:10.1016/j.apjo.2026.100378)
Supplement: Supplementary file 1 [file mmc1.docx]

**SUPPLEMENTARY MATERIALS**

**Risk factors of primary angle-closure glaucoma: An exposome-wide association study in the China Kadoorie Biobank**

[**Members of the China Kadoorie Biobank collaborative group	2**](#_Toc231308134)

[**Supplementary methods	3**](#_Toc231308135)

[(1)	Elastic-Net	3](#_Toc231308136)

[(2)	XGboost	6](#_Toc231308137)

[**Genetic Validation of the 'Probable PACG' Phenotype using Polygenic Risk Scores	8**](#_Toc231308138)

[**Table S1: Inventory of variables considered for feature selection	11**](#_Toc231308139)

[**Table S2: Distribution of glaucoma subtypes in CKB	17**](#_Toc231308140)

[**Table S3: Number of variables included across baseline, discovery, and validation stages	18**](#_Toc231308141)

[**Table S4: Associations of selected exposures with definite PACG incidence	19**](#_Toc231308142)

[**Table S5: Associations of selected exposures with prob PACG incidence, based on competing risks	22**](#_Toc231308143)

[**Figure S1: Flow chart of the control group in Step 1	25**](#_Toc231308144)

[**Figure S2: Flow chart of the study participant	26**](#_Toc231308145)

[**Figure S3: Associations between continuous exposures and definite PACG incidence	27**](#_Toc231308146)

[**Reference	28**](#_Toc231308147)

## Members of the China Kadoorie Biobank collaborative group

**International Steering Committee:** Junshi Chen, Zhengming Chen (PI), Robert Clarke, Rory Collins, Liming Li (PI), Jun Lv, Richard Peto, Robin Walters.

**International Co-ordinating Centre, Oxford:** Daniel Avery, Maxim Barnard, Derrick Bennett, Ruth Boxall, Ka Hung Chan, Yiping Chen, Zhengming Chen, Charlotte Clarke, Johnathan Clarke; Robert Clarke, Huaidong Du, Ahmed Edris Mohamed, Hannah Fry, Simon Gilbert, Pek Kei Im, Andri Iona, Maria Kakkoura, Christiana Kartsonaki, Kshitij Kolhe, Hubert Lam, Kuang Lin, James Liu, Mohsen Mazidi, Iona Millwood, Sam Morris, Qunhua Nie, Alfred Pozarickij, Maryam Rahmati, Paul Ryder, Dan Schmidt, Becky Stevens, Iain Turnbull, Robin Walters, Baihan Wang, Lin Wang, Neil Wright, Ling Yang, Xiaoming Yang, Pang Yao.

**National Co-ordinating Centre, Beijing:** Jun Lv, Canqing Yu, Dianjianyi Sun, Yuanjie Pang, Can Hou, Qingmei Xia, Chao Liu, Pei Pei, Lang Pan, Xiao Han, Honglu Bian, Xinxin Chen.

**10 Regional Co-ordinating Centres:**

Qingdao CDC: Zengchang Pang, Ruqin Gao, Shanpeng Li, Haiping Duan, Shaojie Wang, Yongmei Liu, Ranran Du, Liang Cheng, Xiaocao Tian, Hua Zhang. Licang CDC: Dan Hu, Xiaoyan Zheng, Yujie Wang. Heilongjiang Provincial CDC: Wei Sun, Shichun Yan, Xiaoming Cui. Nangang CDC: Chi Wang, Zhenyuan Wu, Lishun Zhai, Zhaoxi Pang, Shiwen Dong. Hainan Provincial CDC: Huiming Luo, Jinyan Chen, Bin He, Dingwei Sun, Xingren Wang, Tingting Ou. Meilan CDC: Xiangyang Zheng, Dewei Zheng, Shuai Yang, Yilei Li, Lihui Li, Xingjiao Chen. Jiangsu Provincial CDC: Jinyi Zhou, Ran Tao, Jian Su, Xikang Fan, Zongming Cheng, Yuxiao Huang. Suzhou CDC: Yan Lu, Yujie Hua, Li Xing, Shuxian Wang, Jianrong Jin, Juping Ma, Jinchao Liu, Kaifei Zhu, Hongfu Ren, Xingfeng Shen. Guangxi Provincial CDC: Ge Zhong, Wei Mao, Zhenzhen Lu, Ling He. Liuzhou CDC: Lifang Zhou, Changping Xie, Jian Lan, Tingping Zhu, Jinxue Tan, Liuping Wei, Liyuan Zhou, Sisi Wang. Sichuan Provincial CDC: Xianping Wu, Ningmei Zhang, Xiaofang Chen, Xiaoyu Chang, Zhuo Wang, Yujin He. Pengzhou CDC: Mingqiang Yuan, Xia Wu, Xiaofang Chen, Zhaodong Wang, Qiang Sun, Yang Lin. Gansu Provincial CDC: Faqing Chen, Xiaolan Ren, Lijun Chang, Feiming Zhong. Maiji CDC: Jianjun Feng, Weijie Hu, Xiaofang Zhang, Yalin Chen, Fei Wang, Jun Wang. Henan Provincial CDC: Linqi Diao, Wanshen Guo, Zhiwei Han, Dongyang Zhao, Dengjun Zhu, Kai Kang, Shixian Feng, Huizi Tian, Yali Yan, Bing Han, Li Gao, Shaofang Li, Huafei Feng, Wei Tang. Huixian CDC: Xiaolin Li, Huarong Sun, Xiaocong Zhao, Ying Li, Chen Hu, Pan He, Xukui Zhang, Yuanyuan Jin, Hesheng Zhang. Zhejiang Provincial CDC: Min Yu, Ruying Hu, Hao Wang, Weiwei Gong, Jieming Zhong, Meng Wang, Chunxiao Xu, Keqing Gong. Tongxiang CDC: Hao Xu, Yuan Cao, Kaixu Xie, Lingli Chen, Xiaomei Tu,Chen Chen. Hunan Provincial CDC: Xiaojun Li, Li Yin, Huilin Liu, Yuan Liu, Yi Liu, Lei Yin, Xian Xie, Jing Wang. Liuyang CDC: Bo Xiao, Pingsheng Lou, Yuan Peng, Libo Zhang, Chan Qu, Qili Jiang, Yanling Chen, Yan Zhao.

## Supplementary methods

**Initial variable selection**

## (1) Elastic-Net

To perform variable selection, we employed the Elastic-Net method using a logistic regression model and the "glmnet" package in R. We included all potential risk factors as independent variables and glaucoma status as the dependent variable. A bootstrap procedure with 100 resampling iterations was conducted to enhance the stability and generalizability of the variable selection process:

- For each bootstrap sample, we performed 10-fold cross-validation to identify the optimal regularization parameter (λ) that minimized the cross-validation error.
- The Elastic-Net logistic regression model was then fitted to each resampled dataset using the optimal λ value.
- We recorded the variables with non-zero coefficients in each model of the bootstrap samples, and these variables were considered selected.
- After completing all 100 bootstrap samples, we calculated the selection frequency for each variable, defined as the percentage of samples in which the variable was selected.

Finally, we ranked the variables based on their selection frequency, which ranged from 0% to 100%. Variables with a selection frequency exceeding 80% across the 100 bootstrap samples were classified as selected features.

**Figure: The process of Elastic-Net selection**

**Figure: Selection frequency of potential risk factors with non-zero coefficients**

**Figure: Spearman correlation matrix for the selected category variables**

## (2) XGboost

In a parallel statistical analysis strategy, we applied the XGboost method (“xgboost”, “caret”, “rBayesianOptimization”, “SHAPforxgboost” in R packages). XGboost is known for its strong performance on high-dimensional data and its ability to effectively identify key factors highly correlated with the outcome among a large set of variables. Compared to traditional linear models, XGboost can also capture non-linear relationships between variables, accommodating complex interactions of disease factors:

- The data were randomly split into a training set (80%) and a validation set (20%). The training data were shuffled to prevent the sample order from influencing model construction.
- Bayesian optimization was used to identify the optimal hyperparameter combination for the XGboost model, including maximum tree depth, learning rate, gamma, sampling rate, feature subsampling rate, and minimum child weight, to enhance model performance.
- An evaluation function was defined, using the Area Under the Curve (AUC) as the performance metric. The model’s performance across different hyperparameter combinations was assessed using 10-fold cross-validation.
- Initial random search and Bayesian optimization were performed for 100 iterations to obtain the optimal hyperparameter combination.
- After obtaining the best parameters, the final XGboost model was built based on the training set and validated on the validation set. The AUC of the predictions on the validation set was used to assess the model’s predictive performance.

Feature importance scores were extracted, and the top 85% of features, based on the cumulative contribution of their Gain values, were identified as important variables. Additionally, SHAP (Shapley Additive Explanations) values were calculated, and beeswarm plots were generated to visualize the importance of these features and their impact on the model's output.

**Figure: The SHAP beeswarm plots of XGboost selection**

## Genetic Validation of the 'Probable PACG' Phenotype using Polygenic Risk Scores

(unpublished. Charles Li et al. Incidence patterns and genetic validation of primary glaucoma subtypes among 1 million adults in China and the UK)

A subset of approximately 100,000 participants in the China Kadoorie Biobank (CKB) underwent genome-wide genotyping using custom-designed arrays (Walters et al., 2023). For the genetic validation analysis, genotyped participants were included if they had non-missing genetic principal components (PCs) and were eligible for primary glaucoma analysis (including 1,658 total incident primary glaucoma participants: 505 PACG participants and 1,153 unspecified glaucoma participants).

To provide genetic support for the 'probable PACG' subtype inference, polygenic risk scores (PRSs) were derived from ancestry- and subtype-specific genome-wide association study (GWAS) summary statistics. Four PRSs were generated based on summary statistics from: (i) a multi-trait analysis of POAG and related endophenotypes (intraocular pressure and vertical cup-to-disc-ratio) in European populations (MTAG-EUR), (ii) POAG in Europeans (POAG-EUR), (iii) POAG in East Asians (POAG-EAS), and (iv) a newly meta-analysed East Asian PACG dataset (PACG-EAS) combining summary statistics from 2,537 PACG cases and 6,289 controls across mainland China, Hong Kong, Japan, Korea, and Singapore from Khor et al. (2016).

PRSs were constructed using PRS-CSx to infer posterior single nucleotide polymorphism (SNP) effect sizes under a Bayesian continuous shrinkage framework while accounting for ancestry-specific linkage disequilibrium (LD) panels, restricted to HapMap3 variants. Standardised PRSs (z-scores with mean 0 and SD 1) were computed using PLINK v2.00.

Associations between the PRSs and incident glaucoma phenotypes in CKB were examined using multivariable logistic regression, estimating odds ratios (ORs) per SD increase in PRS. Models were adjusted for baseline age, sex, and the first 11 genome-wide national genetic PCs to control for population stratification. The proportion of disease liability explained by each PRS was quantified using the incremental coefficient of determination on the liability scale (Lee ${\Delta R}^{2}$ ), incorporating published population prevalence estimates for liability scale transformation.

**Figure: Polygenic risk score associations with incident glaucoma phenotypes across CKB**

Odds ratios per standard deviation increase in polygenic risk score (PRS) are shown for incident glaucoma phenotypes in CKB, estimated using multivariable logistic regression models adjusted for baseline age, sex, and genetic principal components (11 in CKB).

**Table Incremental liability-scale variance explained by glaucoma polygenic risk scores**

| **PRS Name** | **Phenotype** | **Nagelkerke** $\boldsymbol{\Delta}\boldsymbol{R}^{\boldsymbol{2}}$ | **Population Prevalence (**$\boldsymbol{K}$**)** | **Sample Prevalence (**$\boldsymbol{P}$**)** | **Lee** $\boldsymbol{\Delta}\boldsymbol{R}^{\boldsymbol{2}}$ | **95% LCI** | **95% UCI** |
| --- | --- | --- | --- | --- | --- | --- | --- |
| **MTAG EUR** | PACG | 0.0032048 | 0.0081 | 0.000996185 | 0.423 | 0.009 | 1.573 |
|  | Unspecified Glaucoma | 0.0024620 | 0.0081 | 0.002556197 | 0.127 | 0.010 | 0.402 |
|  | Probable PACG | 0.0029408 | 0.0081 | 0.003547297 | 0.109 | 0.022 | 0.276 |
| **PACG EAS** | PACG | 0.0050547 | 0.0081 | 0.000996185 | 0.668 | 0.131 | 1.693 |
|  | Unspecified Glaucoma | 0.0075178 | 0.0081 | 0.002556197 | 0.388 | 0.130 | 0.758 |
|  | Probable PACG | 0.0073924 | 0.0081 | 0.003547297 | 0.275 | 0.127 | 0.497 |
| **POAG EAS** | PACG | 0.0000029 | 0.0081 | 0.000996185 | 0.000 | 0.000 | 0.368 |
|  | Unspecified Glaucoma | 0.0000135 | 0.0081 | 0.002556197 | 0.001 | 0.000 | 0.080 |
|  | Probable PACG | 0.0000103 | 0.0081 | 0.003547297 | 0.000 | 0.000 | 0.045 |
| **POAG EUR** | PACG | 0.0041274 | 0.0081 | 0.000996185 | 0.545 | 0.027 | 1.822 |
|  | Unspecified Glaucoma | 0.0006002 | 0.0081 | 0.002556197 | 0.031 | 0.000 | 0.185 |
|  | Probable PACG | 0.0014422 | 0.0081 | 0.003547297 | 0.054 | 0.003 | 0.169 |

95% confidence intervals of incremental coefficients of determination on the liability scale (Lee $\Delta R^{2}$) for each polygenic risk score (PRS) and outcome were estimated by nonparametric bootstrap. $\Delta R^{2}$ was computed by first estimating the incremental Nagelkerke pseudo-$R^{2}$ attributable to the PRS from nested logistic regression models (PRS + covariates vs. covariates only), and then transforming this quantity to the liability scale following Lee et al. (2012) to account for case-control ascertainment. Bootstrap confidence intervals were obtained by resampling individuals with replacement (500 replicates), refitting the nested models in each replicate, recomputing incremental Nagelkerke $R^{2}$, and applying the liability-scale transformation to each replicate. The reported interval corresponds to the 2.5th and 97.5th percentiles of the bootstrap distribution. Population prevalence (*K*) and sample case fraction (*P*) values used for the liability-scale transformation are specified. P was calculated from the analysis dataset (and recomputed within each bootstrap replicate). *K* was obtained from published epidemiological estimates; namely, the estimate of PACG prevalence in China was obtained from Shan et al. (2024), and estimates of PACG and POAG prevalence in Europe were obtained from Stuart et al. (2025).

Abbreviations: PRS (Polygenic Risk Score), CKB (China Kadoorie Biobank), MTAG (Multi-Trait Analysis of GWAS), PACG (Primary Angle Closure Glaucoma), POAG (Primary Open Angle Glaucoma), EUR (European), EAS (East Asian), LCI (Lower Confidence Interval), UCI (Upper Confidence Interval).

## Table S1: Inventory of variables considered for feature selection

| **Categories (N)** | **Variable names** | **Definitions** |
| --- | --- | --- |
| Sociodemographic (12) | Sex | 0= Men |
|  |  | 1= Women |
|  | Age | Continuous variable (unit: year)/ Decile categories |
|  | Highest education | 0= No formal school |
|  |  | 1= Primary school |
|  |  | 2= Middle school |
|  |  | 3= High school |
|  |  | 4= College degree or above |
|  | Occupation | 0= Agriculture worker |
|  |  | 1= Factory worker |
|  |  | 2= Technical Personnel |
|  |  | 3= Retired |
|  |  | 4= Unemployed |
|  | Marital status | 0= Married |
|  |  | 1= Others |
|  | Annual household income | 0= <5,000 yuan |
|  |  | 1= 5,000-10,000 yuan |
|  |  | 2= 10,000-20,000 yuan |
|  |  | 3= 20,000-35,000 yuan |
|  |  | 4= ≥35,000 yuan |
|  | Health care cover | 0= No |
|  |  | 1= Yes |
|  | Own house/apartment | 0= No |
|  |  | 1= Yes |
|  | Toilet or private use | 0= No |
|  |  | 1= Yes |
|  | Telephone or mobile phone | 0= No |
|  |  | 1= Yes |
|  | Own motor vehicle | 0= No |
|  |  | 1= Yes |
|  | Holiday during last five years | 0= No |
|  |  | 1= Yes |
| Tea drinking (5) | Frequency | 0= Never |
|  |  | 1= Occasionally |
|  |  | 2= Weekly |
|  | Daily tea consumption | Continuous variable (unit: grams) |
|  | Tea type | 0= Green tea |
|  |  | 1= Oolong tea |
|  |  | 2= Black tea |
|  |  | 3= Others |
|  |  | 4= NA |
|  | Strength | 0= Weak |
|  |  | 1= Moderate |
|  |  | 2= Strong |
|  |  | 3= NA |
|  | Temperature | 0= Room temperature/ warm |
|  |  | 1= Hot |
|  |  | 2= Burning hot |
|  |  | 3= NA |
| Alcohol consumption (5) | Frequency | 0= Never |
|  |  | 1= Occasionally |
|  |  | 2= Ex-regular drinker |
|  |  | 3= Weekly |
|  | Alcohol consumption (typical day) | Continuous variable (unit: grams) |
|  | Daily alcohol intake risk classification (typical day, WHO classification) | 0= Low risk (Men: 1-40g, Women: 1-20g) |
|  |  | 1= Moderate risk (Men: 41-60g, Women: 21-40g) |
|  |  | 2= High risk (Men: >61g, Women: >41g) |
|  |  | 3= NA |
|  | Alcohol flush reaction | 0= Absent |
|  |  | 1= Present |
|  |  | 2= NA |
|  | Alcohol type | 0= Beer |
|  |  | 1= Rice wine |
|  |  | 2= Wine/Fruit wine |
|  |  | 3= Spirits (≥50% alcohol) |
|  |  | 4= Spirits (<50% alcohol) |
|  |  | 5= NA |
| Tobacco use history (3) | Frequency | 0= Never smoker |
|  |  | 1= Occasional smoker |
|  |  | 2= Former smoker |
|  |  | 3= Current smoker |
|  | Daily cigarette consumption | Continuous variable (units: cigarettes per day) |
|  | Inhalation depth | 0= Oral cavity |
|  |  | 1= Pharyngeal |
|  |  | 2= Pulmonary |
|  |  | 3= NA |
| Food group and nutrient supplement intake and other diet-related information (20) | *Frequency of consumption of:* | |
|  | Rice | 0= Rarely/Monthly/Never  1= ≥1 days/week  2= Daily |
|  | Wheat |  |
|  | Other staple food |  |
|  | Meat |  |
|  | Poultry |  |
|  | Fish/sea food |  |
|  | Fresh eggs |  |
|  | Fresh vegetables |  |
|  | Soybean products (including soy milk) |  |
|  | Preserved vegetables |  |
|  | Fresh fruit |  |
|  | Dairy products (milk, yogurt) |  |
|  | *Intake of nutritional supplements:* | |
|  | Fish oil/cod liver oil supplement intake | 0= No  1= Yes |
|  | Vitamins |  |
|  | Calcium/iron/zinc |  |
|  | Ginseng |  |
|  | Other herbal products |  |
|  | Food shortage | 0= No |
|  |  | 1= Yes |
|  | Spicy food | 0= Never |
|  |  | 1= 1-2 days/week |
|  |  | 2= 3-5 days/week |
|  |  | 3= Daily |
|  | Cooking oil type | 0= Do not cook |
|  |  | 1= Rapeseed |
|  |  | 2= Peanut |
|  |  | 3= Soybean |
|  |  | 4= Lard |
|  |  | 5= Other |
| Passive smoking and indoor air pollution (4) | Passive smoking | 0= No |
|  |  | 1= Yes |
|  | Cumulative exposure: average daily duration * years of cohabitation | Continuous variable (unit: hours) |
|  | Air pollution (Heating or Cooking) | 0= No pollution |
|  |  | 1= Using coal/firewood |
|  |  | 2= Other |
|  | Have been coal-smoky? | 0= Never |
|  |  | 1= Yes |
| Personal and family medical history (37) | Self-rated health | 0= Excellent |
|  |  | 1= Good |
|  |  | 2= Fair |
|  |  | 3= Poor |
|  | *Baseline prevalent diseases of:* | |
|  | Diabetes | 0= No  1= Yes |
|  | CHD |  |
|  | Stroke or TIA |  |
|  | Hypertension |  |
|  | Rheumatic heart disease |  |
|  | TB |  |
|  | Emphysema/bronchitis |  |
|  | Asthma |  |
|  | Cirrhosis/Chronic hepatitis |  |
|  | Peptic ulcer |  |
|  | Gallstone/gallbladder disease |  |
|  | Kidney disease |  |
|  | Fracture |  |
|  | Rheumatoid arthritis |  |
|  | Psychiatric disorders |  |
|  | Neurasthenia |  |
|  | Head injury |  |
|  | Cancer |  |
|  | COPD |  |
|  | *Self-reported medication history of:* | |
|  | Aspirin | 0= No  1= Yes |
|  | ACE-I |  |
|  | Beta-blocker |  |
|  | Statins |  |
|  | Diuretics |  |
|  | Ca++ antagonist |  |
|  | Chlorpropamide or metformin |  |
|  | Insulin |  |
|  | Received blood transfusion | 0= None |
|  |  | 1= Once |
|  |  | 2= More than once |
|  | Paid blood donation for financial reasons | 0= No |
|  |  | 1= Yes |
|  | Bowel frequency | 0= >1/day |
|  |  | 1= 1.day |
|  |  | 2= Every other day |
|  |  | 3= <3/week |
|  | Longevity of parents | 0= No |
|  |  | 1= Yes |
|  | *Family history of diseases of:* | |
|  | Stroke | 0= No  1= Yes |
|  | Heart attack |  |
|  | Diabetes |  |
|  | Mental disorder |  |
|  | Cancer |  |
| Physical activities (2) | Daily sedentary leisure time | Continuous variable (unit: hours) / Decile categories |
|  | Total physical activity | Continuous variable (unit: hours/day)/ Decile categories |
| Female reproductive history (5) | First menstrual period | Continuous variable (unit: years) |
|  | Menopause | 0= No |
|  |  | 1= Currently |
|  |  | 2= Yes |
|  | Number of P=pregnancies | Continuous variable (unit: times) |
|  | Use of contraceptive pills | 0= No |
|  |  | 1= Yes |
|  | *Surgical history of:* | |
|  | Hysterectomy | 0= No  1= Yes |
|  | Oophorectomy |  |
|  | Breast lump/tumor |  |
| Sleep patterns, psychological status, and mental health (8) | Life satisfaction | 0= Very/Basically |
|  |  | 1= Neither satisfied nor dissatisfied |
|  |  | 2= Unsatisfied/Very unsatisfied |
|  | *Any negative life events:* | |
|  | Marital separation/divorce | 0= No  1= Yes |
|  | Loss of job/retirement |  |
|  | Business bankrupt |  |
|  | Violence |  |
|  | Major conflict within family |  |
|  | Major injury or traffic accident |  |
|  | Death /major illness of spouse |  |
|  | Death/major illness of other close family member |  |
|  | Major natural disaster (e.g. flood & drought) |  |
|  | Loss of income / living on debt |  |
|  | *Any sleep problems:* | |
|  | Taking >30 minutes to fall asleep after going to bed or waking up in the middle of the night | 0= No  1= Yes |
|  | Waking up early and not being able to go back to sleep |  |
|  | Needing to take medicine (including herbal or sleeping pills) at least once a week to help sleep |  |
|  | Having difficulty staying alert while at work, eating or meeting people during daytime |  |
|  | Daytime napping | 0= No |
|  |  | 1= Yes |
|  | Snoring | 0= No |
|  |  | 1= Yes |
|  | Sleep duration | Continuous variable (unit: hours) / Decile categories |
|  | Depressive symptoms (CIDI-A) | 0= No |
|  |  | 1= Yes |
|  | Anxiety symptoms (CIDI-B) | 0= No |
|  |  | 1= Yes |
| Physical examination (14) | Standing height | Continuous variable (unit: cm) / Decile categories |
|  | Sitting height | Continuous variable (unit: cm) / Decile categories |
|  | Body mass index | Continuous variable (unit: kg/m²)/ Decile categories |
|  | Waist | Continuous variable (unit: cm) / Decile categories |
|  | Hip | Continuous variable (unit: cm) / Decile categories |
|  | Waist-to-hip ratio | Continuous variable/ Decile categories |
|  | Waist-to-height Ratio | Continuous variable/ Decile categories |
|  | Body fat percentage | Continuous variable (%)/ Decile categories |
|  | Systolic blood pressure | Continuous variable (unit: mmHg)/ Decile categories |
|  | Diastolic blood pressure | Continuous variable (unit: mmHg) / Decile categories |
|  | Heart rate | Continuous variable (unit: bpm) / Decile categories |
|  | CO | Continuous variable (unit: ppm) / Decile categories |
|  | %COHB | Continuous variable (%)/ Decile categories |
|  | Lung function (FEV1/ FVC) | Continuous variable/ Decile categories |
| Blood test (2) | Hepatitis B test | 0= Negative |
|  |  | 1= Positive |
|  |  | 2= Missing |
|  | Randomly blood glucose | Continuous variable (unit: mmol/L)/ Decile categories |

## Table S2: Distribution of glaucoma subtypes in CKB

| **ICD10 and description** | **Participants with glaucoma in CKB** |
| --- | --- |
| H40.0-Glaucoma suspect  (Ocular hypertension) | 124 (2.6%) |
| H40.1-POAG | 174 (3.6%) |
| H40.2-PACG | 1,372 (28.7%) |
| H40.3-H40.6 (Secondary glaucoma) | 645 (13.5%) |
| H40.8-Other glaucoma | 35 (0.7%) |
| H40.9-Unspecified glaucoma | 2,432 (50.9%) |

## Table S3: Number of variables included across baseline, discovery, and validation stages

| **Category** | **Baseline**  **(N= 117)** | **Discovery^*^** | | **Validation**  **(N= 26)** |
| --- | --- | --- | --- | --- |
|  |  | **Elastic-Net-selected**  **(N= 84)** | **XGboost-selected**  **(N= 31)** |  |
| Sociodemographics | 12 | 9 | 5 | 7 |
| Tea drinking | 5 | 4 | 1 | 1 |
| Alcohol | 5 | 3 | 0 | 0 |
| Tobacco | 3 | 2 | 0 | 0 |
| Diet | 20 | 19 | 6 | 6 |
| Passive smoking and indoor air pollution | 4 | 1 | 1 | 0 |
| Medical history | 37 | 24 | 2 | 3 |
| Mental health | 8 | 5 | 1 | 1 |
| Physical activities | 2 | 2 | 1 | 1 |
| Reproductive history | 5 | 1 | 1 | 1 |
| Physical examination | 14 | 12 | 12 | 5 |
| Blood tests | 2 | 2 | 1 | 1 |

*A total of 86 unique exposures were identified across both Elastic-Net and XGboost, all of which were subsequently considered for validation analyses.

## Table S4: Associations of selected exposures with definite PACG incidence (n=1,015)

| **Categories** | **HR (95% CI)** | **P** |
| --- | --- | --- |
| **Sociodemographic groups** |  |  |
| **Age** |  | <0.001 |
| per year | 1.07 (1.06 - 1.08) |  |
| **Sex: Women** | 1.83 (1.58 - 2.13) | <0.001 |
| **Education** |  |  |
| No formal school | Ref. |  |
| Primary school | 0.94 (0.78 - 1.14) | 0.53 |
| Middle school | 0.81 (0.64 - 1.01) | 0.06 |
| High school | 0.68 (0.52 - 0.88) | 0.01 |
| College or above | 0.82 (0.61 - 1.14) | 0.24 |
| **Occupation** |  |  |
| Agriculture worker | Ref. |  |
| Factory worker | 1.34 (0.99 – 1.82) | 0.06 |
| Technical personnel | 1.50 (1.22 - 1.85) | <0.001 |
| Retired | 1.60 (1.24 - 2.07) | <0.001 |
| Unemployed | 1.46 (0.99 – 2.14) | 0.05 |
| **Annual household income (yuan)** |  |  |
| <5,000 | Ref. |  |
| 5,000-10,000 | 1.30 (0.97 - 1.75) | 0.08 |
| 10,000-20,000 | 1.47 (1.10 - 1.95) | 0.01 |
| 20,000-35,000 | 1.38 (1.02 - 1.87) | 0.04 |
| >=35,000 | 1.56 (1.13 – 2.15) | <0.001 |
| **Health care cover** | Ref. | 0.01 |
| No |  |  |
| Yes | 1.33 (1.10 - 1.60) |  |
| **Diet groups** |  |  |
| **Cooking oil** |  |  |
| Do not cook | Ref. |  |
| Rapeseed | 1.01 (0.77 - 1.32) | 0.94 |
| Peanut | 1.14 (0.89 - 1.46) | 0.29 |
| Soybean | 0.89 (0.67 - 1.19) | 0.45 |
| Lard | 0.97 (0.71 - 1.33) | 0.87 |
| Other | 0.98 (0.76 - 1.27) | 0.88 |
| **Diet** |  |  |
| **Dairy products** |  |  |
| Rarely/monthly/never | Ref. |  |
| >=1 days/week | 1.06 (0.87 - 1.29) | 0.54 |
| Daily | 1.17 (1.01 - 1.39) | 0.04 |
| **Preserved vegetables** |  |  |
| Rarely/monthly/never | Ref. |  |
| >=1 days/week | 0.96 (0.81 - 1.12) | 0.59 |
| Daily | 1.14 (0.94 - 1.38) | 0.18 |
| **Soybean** |  |  |
| Rarely/monthly/never | Ref. |  |
| >=1 days/week | 1.00 (0.86 - 1.16) | 0.10 |
| Daily | 0.91 (0.71 - 1.15) | 0.43 |
| **Nutritional Supplements** |  |  |
| **Ginseng** |  | 0.01 |
| No | Ref. |  |
| Yes | 1.73 (1.12 - 2.65) |  |
| **Other herbal products** |  | 0.11 |
| No | Ref. |  |
| Yes | 1.18 (0.96 - 1.44) |  |
| **Health behaviours groups** |  |  |
| **Physical activity** |  |  |
| Q1 | Ref. |  |
| Q2 | 1.10 (0.95 - 1.29) | 0.21 |
| Q3 | 0.88 (0.71 - 1.09) | 0.24 |
| Q4 | 0.95 (0.75 - 1.21) | 0.68 |
| **Tea consumption** |  |  |
| Never | Ref. |  |
| Occasionally | 0.93 (0.79 - 1.09) | 0.37 |
| Weekly | 0.95 (0.79 - 1.15) | 0.60 |
| **Alcohol consumption** |  |  |
| Never | Ref. |  |
| Occasionally | 1.04 (0.89 - 1.22) | 0.59 |
| Ex-regular | 1.18 (0.82 - 1.69) | 0.37 |
| Weekly | 1.00 (0.77 - 1.30) | 0.10 |
| **Tobacco use** |  |  |
| Never | Ref. |  |
| Occasionally | 0.93 (0.66 - 1.32) | 0.69 |
| Former | 0.81 (0.57 - 1.15) | 0.25 |
| Current | 0.88 (0.68 - 1.14) | 0.33 |
| **Medical history groups** |  |  |
| **Self-rated health** |  |  |
| Excellent | Ref. |  |
| Good | 1.18 (0.96 - 1.45) | 0.11 |
| Fair | 1.14 (0.94 - 1.38) | 0.18 |
| Poor | 1.06 (0.81 - 1.39) | 0.65 |
| **Sleep problem** |  |  |
| No | Ref. | 0.30 |
| Yes | 1.09 (0.93 - 1.27) |  |
| **Menopause status (women)** |  |  |
| No | Ref. |  |
| Currently | 1.16 (0.76 - 1.78) | 0.49 |
| Yes | 1.60 (1.16 - 2.19) | 0.01 |
| **Baseline prevalent diseases** |  |  |
| **Diabetes** |  |  |
| No | Ref. | 0.04 |
| Yes | 1.21 (1.00 - 1.52) |  |
| **Peptic ulcer** |  |  |
| No | Ref. | 0.03 |
| Yes | 1.31 (1.01 - 1.73) |  |
| **Hypertension** |  |  |
| No | Ref. | 0.15 |
| Yes | 0.91 (0.79 – 1.04) |  |

Definite PACG (ICD10: H40.2) as the outcome. HR= Hazard ratio; Ref= Reference; PA= Physical activity: The quartiles of physical activity were determined separately for men and women. Model adjustments were performed using a stepwise regression strategy Model adjustments were performed using a stepwise regression strategy. For sociodemographic factors, Model 1 was adjusted for age, sex, region, education, occupation, marital status, household income, and healthcare coverage. Model 2 additionally adjusted for dietary factors and retained significant variables as the final model 2. Model 3 additionally adjusted for health behavior factors and retained significant variables as the final model 3. Finally, Model 4 additionally adjusted for medical history factors and retained significant variables as the final model (Model 1= age+ sex+ region+ education+ occupation+ marital status+ household income+ healthcare coverage; Model 2= Model1+ dairy+ preserved vegetables+ soybean+ ginseng+ herbs+ cooking oil; Model 3= Model2+ pa+ tea+ smoking+ drinking; Model4= Model3+ sleep problem+ self-rated health+ diabetes+ peptic ulcer+ hypertension).

## Table S5: Associations of selected exposures with prob PACG incidence, based on competing risks

|  | **HR (95%CI)** | **P** |
| --- | --- | --- |
| **Sociodemographic groups** |  |  |
| **Age** |  |  |
| per year | 1.01 (1.01 - 1.02) | 0.04 |
| **Sex: Women** | 1.75 (1.62 - 1.90) | <0.001 |
| **Education** |  |  |
| No formal school | Ref. |  |
| Primary school | 0.95 (0.85 - 1.06) | 0.34 |
| Middle school | 0.97 (0.85 - 1.10) | 0.59 |
| High school | 0.91 (0.78 - 1.05) | 0.20 |
| College or above | 0.93 (0.77 - 1.11) | 0.41 |
| **Occupation** |  |  |
| Agriculture worker | Ref. |  |
| Factory worker | 1.10 (0.93 – 1.30) | 0.28 |
| Technical personnel | 1.10 (0.98 - 1.24) | 0.11 |
| Retired | 1.31 (1.13 - 1.51) | <0.001 |
| Unemployed | 1.22 (0.98 - 1.53) | 0.07 |
| **Annual household income (yuan)** |  |  |
| <5,000 | Ref. |  |
| 5,000-10,000 | 1.19 (1.01 - 1.39) | 0.03 |
| 10,000-20,000 | 1.26 (1.08 - 1.47) | <0.001 |
| 20,000-35,000 | 1.34 (1.14 - 1.58) | <0.001 |
| >=35,000 | 1.26 (1.06 - 1.50) | 0.01 |
| **Health care cover** |  |  |
| No | Ref. |  |
| Yes | 1.25 (1.12 - 1.39) | <0.001 |
| **Diet groups** |  |  |
| **Cooking oil** |  |  |
| Do not cook | Ref. |  |
| Rapeseed | 1.05 (0.89 - 1.23) | 0.58 |
| Peanut | 1.23 (1.07 - 1.42) | 0.01 |
| Soybean | 1.07 (0.92 - 1.24) | 0.41 |
| Lard | 1.10 (0.92 - 1.31) | 0.31 |
| Other | 1.19 (1.03 - 1.38) | 0.02 |
| **Diet** |  |  |
| **Dairy products** |  |  |
| Rarely/monthly/never | Ref. |  |
| >=1 days/week | 1.09 (0.97 - 1.21) | 0.14 |
| Daily | 1.14 (1.04 - 1.25) | 0.01 |
| **Preserved vegetables** |  |  |
| Rarely/monthly/never | Ref. |  |
| >=1 days/week | 1.08 (0.99 - 1.18) | 0.08 |
| Daily | 1.15 (1.03 - 1.27) | 0.01 |
| **Soybean** |  |  |
| Rarely/monthly/never | Ref. |  |
| >=1 days/week | 0.95 (0.87 - 1.04) | 0.26 |
| Daily | 0.88 (0.77 - 1.00) | 0.05 |
| **Nutritional Supplements** |  |  |
| **Ginseng** |  |  |
| No | Ref. |  |
| Yes | 1.51 (1.16 - 1.97) | <0.001 |
| **Other herbal products** |  |  |
| No | Ref. |  |
| Yes | 1.16 (1.04 - 1.29) | 0.01 |
| **Health behaviours groups** |  |  |
| **Physical activity** |  |  |
| Q1 | Ref. |  |
| Q2 | 1.01 (0.93 - 1.10) | 0.81 |
| Q3 | 0.98 (0.87 - 1.10) | 0.73 |
| Q4 | 0.93 (0.84 - 1.11) | 0.59 |
| **Tea consumption** |  |  |
| Never | Ref. |  |
| Occasionally | 0.92 (0.84 - 1.00) | 0.04 |
| Weekly | 0.88 (0.79 - 0.98) | 0.02 |
| **Alcohol consumption** |  |  |
| Never | Ref. |  |
| Occasionally | 0.99 (0.91 - 1.08) | 0.79 |
| Ex-regular | 1.07 (0.88 - 1.29) | 0.52 |
| Weekly | 1.07 (0.94 - 1.23) | 0.31 |
| **Tobacco use** |  |  |
| Never | Ref. |  |
| Occasionally | 0.99 (0.82 - 1.19) | 0.89 |
| Former | 0.88 (0.74 - 1.05) | 0.16 |
| Current | 0.85 (0.74 – 0.97) | 0.02 |
| **Medical history groups** |  |  |
| **Self-rated health** |  |  |
| Excellent | Ref. |  |
| Good | 1.13 (1.01 - 1.27) | 0.03 |
| Fair | 1.14 (1.03 - 1.26) | 0.02 |
| Poor | 1.10 (0.96 - 1.27) | 0.17 |
| **Sleep problem** |  |  |
| No | Ref. |  |
| Yes | 1.07 (1.00 - 1.17) | 0.04 |
| **Menopause status (women)** |  |  |
| No | Ref. |  |
| Currently | 0.96 (0.74 - 1.25) | 0.77 |
| Yes | 1.24 (1.02 - 1.50) | 0.03 |
| **Baseline prevalent diseases** |  |  |
| **Diabetes** |  |  |
| No | Ref. |  |
| Yes | 1.27(1.14 - 1.42) | <0.001 |
| **Peptic ulcer** |  |  |
| No | Ref. |  |
| Yes | 1.28 (1.09 - 1.50) | <0.001 |
| **Hypertension** |  |  |
| No | Ref. |  |
| Yes | 0.95 (0.88 - 1.02) | 0.17 |

HR= Hazard ratio; Ref= Reference; PA= Physical activity: The quartiles of physical activity were determined separately for men and women. For sociodemographic factors, Model 1 was adjusted for age, sex, region, education, occupation, marital status, household income, and healthcare coverage. Model 2 additionally adjusted for dietary factors and retained significant variables as the final model 2. Model 3 additionally adjusted for health behavior factors and retained significant variables as the final model 3. Finally, Model 4 additionally adjusted for medical history factors and retained significant variables as the final model (Model 1= age+ sex+ region+ education+ occupation+ marital status+ household income+ healthcare coverage; Model 2= Model 1+ dairy+ preserved vegetables+ soybean+ ginseng+ herbs+ cooking oil; Model 3= Model 2+ pa+ tea+ smoking+ drinking; Model 4= Model 3+ sleep problem+ self-rated health+ diabetes+ peptic ulcer+ hypertension).

## Figure S1: Flow chart of the control group in Step 1

^*^ Missing data referred to any missing values for BMI, random plasma glucose or body fat percentage. Outliers were identified using an Isolation Forest model^1^, which flagged data points with high anomaly scores. The model was trained on all continuous variables, including age, sitting hours, MET, sleep hours, standing height, sitting height, BMI, waist circumference, hip circumference, SBP, DBP, and random plasma glucose.

## Figure S2: Flow chart of the study participant

^*^Missing data (n=8,580) referred to any missing values for BMI (n=2), random plasma glucose (n=8,341) or body fat percentage (n=240).

^†^Outliers were identified using an Isolation Forest model^1^, which flagged data points with high anomaly scores. The model was trained on all continuous variables, including age, sitting hours, MET, sleep hours, standing height, sitting height, BMI, waist circumference, hip circumference, SBP, DBP, and random plasma glucose.

## Figure S3: Associations between continuous exposures and definite PACG incidence

HR = hazard ratio; CI = confidence interval; RPG= random plasma glucose; SBP = systolic blood pressure; DBP = diastolic blood pressure; PP = pulse pressure (PP= SBP -DBP); BMI = body mass index. All analyses used restricted cubic splines, and adjusted for age (except for the model, which examined age as the main exposure), sex, region, education, occupation, marital status, household income, healthcare coverage, tea consumption, dairy products, preserved vegetables consumption, soybean intake, tobacco, and alcohol consumption. For blood pressure analyses, additional adjustment was using the residuals of SBP/DBP after regressing DBP on SBP (and vice versa); for adiposity measures, BMI and waist circumference were mutually adjusted.

## Reference

1	Najman K, Zieliński K. Outlier Detection with the Use of Isolation Forests. In: Jajuga K, Najman K, Walesiak M, eds. Data Analysis and Classification. Cham: Springer International Publishing, 2021: 65–79.
